# Structural and Electrical Comparison of Si and Zr Doped Hafnium Oxide Thin Films and Integrated FeFETs Utilizing Transmission Kikuchi Diffraction

**DOI:** 10.3390/nano10020384

**Published:** 2020-02-22

**Authors:** Maximilian Lederer, Thomas Kämpfe, Norman Vogel, Dirk Utess, Beate Volkmann, Tarek Ali, Ricardo Olivo, Johannes Müller, Sven Beyer, Martin Trentzsch, Konrad Seidel, Lukas M. Eng

**Affiliations:** 1 Fraunhofer IPMS, 01109 Dresden, Germany; thomas.kaempfe@ipms.fraunhofer.de (T.K.);; 2 Institut für Angewandte Physik, Technische Universität Dresden, 01069 Dresden, Germany; lukas.eng@tu-dresden.de; 3 GLOBALFOUNDRIES Fab1 LLC & Co. KG, 01109 Dresden, Germanysven.beyer@globalfoundries.com (S.B.);

**Keywords:** ferroelectrics, hafnium oxide, electron backscatter diffraction, transmission electron microscopy, ferroelectric field effect transistor, non-volatile memory

## Abstract

The microstructure of ferroelectric hafnium oxide plays a vital role for its application, e.g., non-volatile memories. In this study, transmission Kikuchi diffraction and scanning transmission electron microscopy STEM techniques are used to compare the crystallographic phase and orientation of Si and Zr doped HfO_2_ thin films as well as integrated in a 22 nm fully-depleted silicon-on-insulator (FDSOI) ferroelectric field effect transistor (FeFET). Both HfO_2_ films showed a predominately orthorhombic phase in accordance with electrical measurements and X-ray diffraction XRD data. Furthermore, a stronger texture is found for the microstructure of the Si doped HfO_2_ (HSO) thin film, which is attributed to stress conditions inside the film stack during crystallization. For the HSO thin film fabricated in a metal-oxide-semiconductor (MOS) like structure, a different microstructure, with no apparent texture as well as a different fraction of orthorhombic phase is observed. The 22 nm FDSOI FeFET showed an orthorhombic phase for the HSO layer, as well as an out-of-plane texture of the [111]-axis, which is preferable for the application as non-volatile memory.

## 1. Introduction

Due to the high coercive field, compatibility with conventional complementary metal-oxide semiconductor (CMOS) processes and persistent ferroelectricity for ultra thin layers [1,2], HfO_2_ shows excellent properties for non-volatile memories such as ferroelectric field effect transistors (FeFET) [3], ferroelectric random-access memories (FeRAM) [4], and ferroelectric tunneling junctions (FTJs) [5].

Ferroelectric properties in HfO_2_, which have been reported to originate from the orthorhombic phase of the space group Pca2_1_ [6], have already been demonstrated in polycrystalline films doped with various elements such as Y, Sr, Al, Si, or Zr [5] as well as in undoped films [7]. Furthermore, epitaxially grown films of ferroelectric HfO_2_ have been recently reported [8,9].

Since the ferroelectric orthorhombic phase of HfO_2_ is only a metastable phase [10], polycrystalline films can contain a multitude of phases. Here, the monoclinic phase of space group P2_1_/c, which is the ground state of bulk HfO_2_, as well as the tetragonal phase (P4_2_/nmc) and the cubic phase (Fm3m) are suggested by density functional theory based calculations to be most likely present [10]. The phase composition as well as associated textures of the film can be influenced by stress [10], doping [5], thermal treatment [11], and film thickness [12]. It should be mentioned here that, except the phase of space group *Pca*2_1_, two other orthorhombic phases, which are of space group Pbca and Pnma respectively, have been reported for HfO_2_ [10]. As the stabilization of these phases requires high pressure and since both phases do not exhibit ferroelectricity, they are not discussed further in this article.

For the application in highly scaled non-volatile memories, such as in 28 nm and 22 nm technology node high-k metal gate (HKMG) CMOS processes where FeFETs using ferroelectric HfO_2_ thin films have already been demonstrated [13,14], the local orientation and crystallographic phase of the HfO_2_ grains and the overall microstructure of the thin film are of vital importance.

In this article, we investigate Si:HfO_2_ (HSO) and Hf_0.5_Zr_0.5_O_2_ (HZO) films utilizing transmission Kikuchi diffraction (TKD), which allows for analyzing the microstructure as well as the local crystallographic phase and orientation of the HfO_2_ film [15], X-ray diffraction (XRD), and electrical characterization. Furthermore, we use scanning transmission electron microscopy (STEM) techniques for mapping the local crystallographic phase and orientation in a HSO based FeFET fabricated in 22 nm fully-depleted silicon-on-insulator (FDSOI) [14] technology.

## 2. Materials and Methods

The samples with a metal-ferroelectric-metal (MFM) structure were prepared by firstly depositing a 10 nm thick TiN bottom electrode on a highly p-doped silicon wafer using atomic layer deposition (ALD). For the HSO layer, HfCl_4_ and SiCl_4_ precursors were deposited in a 20:1 ratio with an ALD process, resulting in a silicon content of approximately 3.6 atm%. Similarly, the ZrCl_4_ precursor was used in a 1:1 ratio for the Hf_0.5_Zr_0.5_O_2_ deposition. The thickness of the layer was in both cases 10 nm. After ALD of the respective ferroelectric layer, physical vapor deposition (PVD) was used for a TiN top electrode, also called capping layer, and a rapid thermal spike annealing process at 800 °C was performed.

Capacitors required for electrical measurements were structured by deposition of metal (Ti/Pt) contacts utilizing a shadow mask and a subsequent SC1 wet etch, to remove the conductive capping layer. Polarization–Voltage (P–V) loops were measured with a peak amplitude of 3 V at 1 kHz using an aixACCT TF Analyzer 3000 measurement setup. For the cycling of the material, the same conditions were applied.

For the structural investigation of the MFM samples, grazing incidence XRD (GIXRD) and TKD were conducted. Former uses a Bruker D8 Discover XRD system, collecting patterns in a 2*θ* range of 10° to 70° at a fixed incident angle of 0.5°. For TKD analysis, the samples were dimpled and the measurements were performed in a scanning electron microscope (SEM) utilizing a Bruker Optimus TKD detector. The applied acceleration voltage was 30 kV. It should be noted here that a thin TiN layer is present on top of the HfO_2_ layer. As the measurement is performed in transmission, this will result in weak artefacts and/or increased noise level in the detector image [15,16].

For the structural analysis of highly scaled embedded non-volatile memory (eNVM) devices, FeFETs were integrated into a 22 nm FDSOI platform using a non-invasive eNVM process [14]. Slightly larger HZO and HSO based FeFETs, which are used for analysis of material influences on the device properties, were prepared in an HKMG CMOS process replacing the dielectric HfO_2_ with HSO or HZO. Furthermore, a SiON interface is used instead of the native oxide.

The maximum memory window (MW), which is defined for a given current, describes the maximum of threshold voltage shift possible for a given device. This shift results from the change in the polarization state of the ferroelectric layer. Here, the program and erase state, which represent the two extrema of the polarization, were written by applying a positive or negative 5 V pulse for 300 ns, respectively. The threshold voltage of each state was extracted at a current of 10 nA from the transfer characteristics of the transistor.

Structural investigation of the cross-section and in-plane section, which was prepared without structuring, were performed using a STEM and a two-dimensional (2D) detector. This allows for measuring the complete diffraction pattern, which is essential for the analysis of crystallographic phase and orientation.

## 3. Results

**Figure 1:**
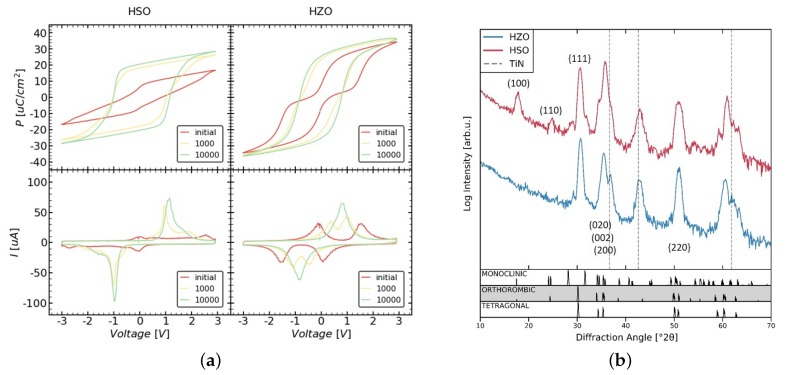
Electrical measurements (**a**) and grazing incident X-ray diffraction (GIXRD) patterns (**b**) of the Si- and Zr-doped HfO_2_ (HSO/HZO) metal-ferroelectric-metal (MFM) sample. (**a**) Polarization–voltage (P–V) as well as the corresponding current–voltage (I–V) loops before and after cycling. Both samples exhibit a pronounced wake-up effect. GIXRD patterns (**b**) show a larger monoclinic phase fraction in case of HSO. Furthermore, differences in the intensity ratio of specific peaks (e.g., (100) and (110)) of the same phase indicate a strongly pronounced texture inside the HSO film.

The electrical measurements of the MFM samples show for both HSO and HZO that an initially pinched P–V loop (see Figure 1a) and thus a anti-ferroelectric-like behavior. With cycling, the loops become more ferroelectric, as can be seen from the resulting merged peaks in the corresponding current–voltage (I–V) loops. This behavior is called wake-up effect.

After wake-up, both samples show a increased remanent polarization (P_r_), but the HSO layer (2P_r_ = 40.68 μC/cm^2^) has a 20% lower 2P_r_ than the HZO layer (2P_r_ = 51.03 μC/cm^2^), while, in case of HZO, the peaks in the corresponding current-voltage (I–V) loops merged to a symmetric peak, HSO shows a very sharp peak in addition to a rather small broad peak. Analogously, the P–V loop appears more square-like. This indicates a pronounced texture or even a favored presence of certain orientation of the polarization axis in the film, since a sharp peak resembles a very small coercive field distribution. The coercive field (2E_c_) extracted from the P–V loops or HSO and HZO is 2.17 V and 1.59 V, respectively. Differences can be explained by texture, which is likely to be present as indicated by the I–V loops.

The measured diffraction patterns of both samples are presented in Figure 1b. HSO as well as HZO show a predominant orthorhombic, tetragonal, or cubic phase. Since all of them show very similar diffraction lines, they are not distinguishable. Furthermore, both samples seem to contain small amounts of the monoclinic phase, as seen by the two diffraction lines close to 30° with intensities near the noise level. Additionally, the HSO sample shows a clear diffraction line at around 17°, which can originate from the orthorhombic as well as monoclinic phase. Since this line corresponds to the [100]-axis and should show much lower intensity than the lines at around 30°, it suggests the presence of strongly pronounced texture or even a preferred crystallographic orientation of the monoclinic and/or orthorhombic grains. This is further supported by the increased intensity of the lines around 35°, which correspond to the <200>-axes. In addition, in the case of HZO, the lines around 35° appear to have an increased intensity, suggesting a texture for this material as well.

**Figure 2:**
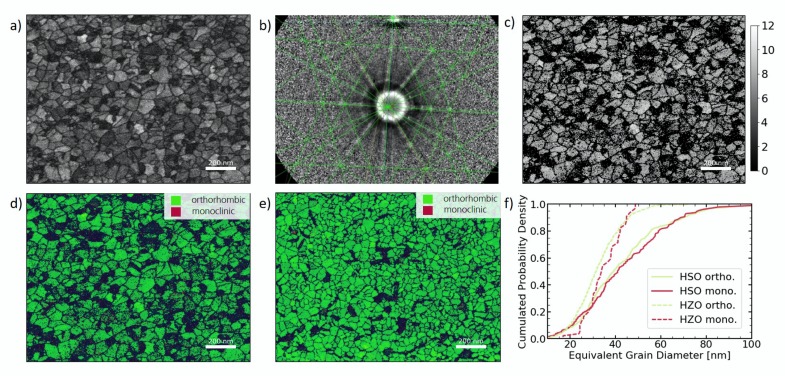
Transmission Kikuchi diffraction analysis of HSO and HZO layers. (**a**) shows the quality map extracted from the scatter signal of HSO film. The measured Kikuchi patterns (here shown for HZO) are fitted with given phases, shown for orthorhombic phase in (**b**) and the reliability of the fit can be estimated from the number of indexed lines, which is visualized in (**c**) for HSO. The assigned phases can be shown in so-called phase maps, shown in (**d**) and (**e**) for HSO and HZO, respectively. The extracted equivalent diameter of the grains is displayed in the form of cumulative distributions (**f**).

While TKD measurements were performed on both MFM samples, the following results are focused on the HSO sample. The HZO sample has been analyzed in the same manner. By visualizing the intensity of the scatter signal for all measured Kikuchi patterns, a so-called quality image can be constructed (see Figure 2a). This visualization allows for already identifying grains and their degree of symmetry, as high symmetric phases appear bright, whereas dark areas resemble low symmetric phases or amorphous regions like grain boundaries.

For given crystal structures, in this case the orthorhombic and monoclinic phase of HfO_2_, the expected Kikuchi patterns can be simulated and fitted to the measured ones (see Figure 2b). From the amount of indexed lines, the trust in each fit can be estimated and visualized (see Figure 2c). By color coding the fitted phases, the crystallographic phase distribution of the microstructure can be visualized as shown for HSO and HZO in Figure 2d,e, respectively. It should be noted here that the tetragonal and cubic phases were not taken into account, since their Kikuchi patterns are quite similar, thus rendering a fit to distinguish these phases much more difficult. Therefore, some tetragonal or cubic grains will be indexed as orthorhombic in the fit.

Figure 2d identifies grains that appeared quite dark in the quality map (Figure 2a) as monoclinic, which appears darker due to its lower symmetry. Since the scatter signal is much weaker for these grains, large amounts of the grain could not be indexed. Nevertheless, it can be seen in Figure 2c that, for points indexed as monoclinic, many lines could still be fitted. Furthermore, the sum of data points for each grain allowed a clear assignment to one of the given phases. Consequently, the grain size distribution can be extracted from Figure 2d,e for each phase and is visualized as cumulative probability distribution in Figure 2f.

**Figure 3:**
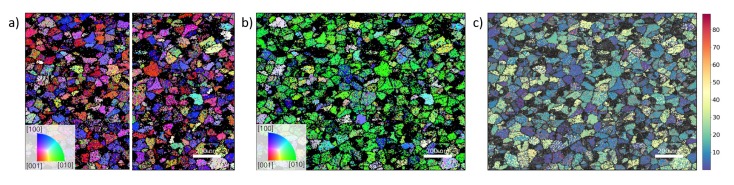
Crystallographic orientation of the grains in the (100)- and (010)-plane of the HSO sample, corresponding to the crystallographic in-plane axes, are visualized in (**a**). Crystallographic axes perpendicular to (001)-plane of the sample, thus resembling out-of-plane orientation, are shown in (**b**). The angle between the sample plane and the polarization axis of orthorhombic HfO_2_ is visualized in (**c**).

Since the crystallographic orientation of each measurement point can be analyzed, TKD also enables the visualization of the microstructures’ orientation distribution. Figure 3a,b show the crystallographic direction of the orthorhombic grains, which were indexed in Figure 2d, in- and out-of-plane of the HSO thin film, respectively. From this, a strong out-of-plane texture of the [010]-axis can already be deduced. Furthermore, Figure 3c, which visualizes the angle of the polarization axis with the sample plane, shows that most of the grains have their polarization [001]-axis lying in-plane.

**Figure 4:**
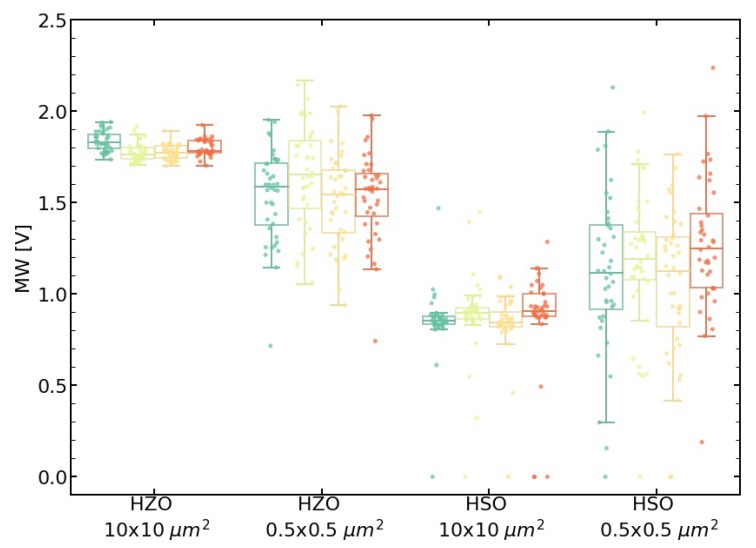
In-die variability of the memory window is smaller for HZO as well as for larger devices. The die-to-die variability of the four dies (visualized by different color) is rather low.

Regarding the influences of the microstructure difference between HSO and HZO on integrated devices, the in-die variability of the memory window was investigated for two device layouts (see Figure 4). For HSO and HZO FeFETs, the larger device (10 × 10 μm^2^) shows a smaller variability. In the case of the small devices (0.5 × 0.5 μm^2^), broken FeFETs also appear with a memory window close to zero. Furthermore, the HSO shows a larger MW distribution than HZO.

**Figure 5:**
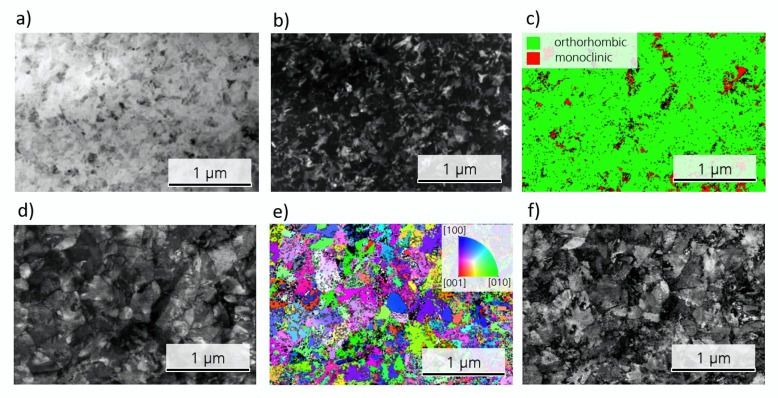
STEM analysis of a planar MFIS HSO layer. (**a**,**b**) shows the bright field and conical dark field, respectively. The extracted phases are visualized in (**c**) and the reliability of their fit is shown in (**d**). Analogously, (**e**,**f**) shows the crystallographic orientation and the reliability of the fit, respectively.

To analyze the present microstructure in highly scaled FeFETs, a planar film (before structuring) and a cross-section of a 22 nm FDSOI FeFET [14] are investigated using STEM with a 2D detector with high dynamic range for each pixel. Like the TKD measurement in an SEM, the analysis is done in transmission, with the detector located inside the beam. The main differences here are the accelerator voltage and an adjustable convergence angle, which allows for tuning the measured signal between classical electron diffraction and Kikuchi diffraction patterns [17]. The former is used in this analysis and can be similar to TKD fitted with given phases. Furthermore, bright field and high angular dark field images can be calculated by integrating the corresponding detector regions, which is shown for the planar film in Figure 5a,b.

From the visualization of the indexed phases (see Figure 5c), it is apparent that the orthorhombic phase is predominant, but some monoclinic grains are still remaining. The crystallographic phase fitting quality is visualized in Figure 5d. In this sample, only weak texture can be observed (see Figure 5e and corresponding fit reliability in Figure 5f).

**Figure 6:**
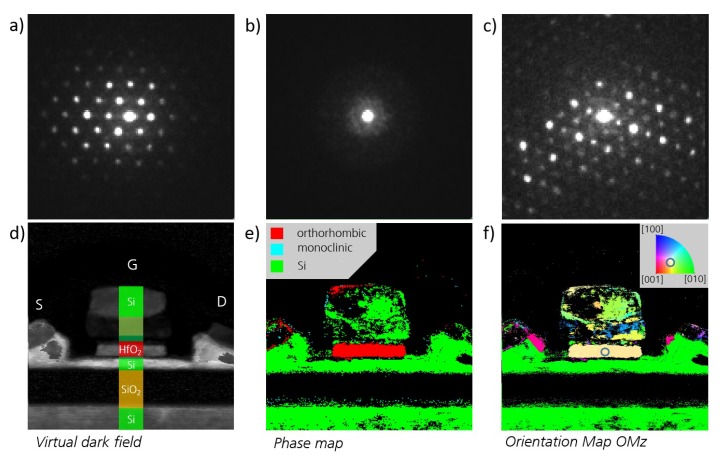
(**a**–**c**) display the measured diffraction pattern for measurement points from the silicon, silicon oxide, and hafnium oxide region, respectively; (**d**) shows the dark field of the cross-section of a 22 nm FDSOI FeFET. The phase map is shown in (**e**), showing predominantly orthorhombic HfO_2_. The crystallographic axis parallel to the gate stack is visualized in (**f**) indicating an orientation close to [111].

In the cross-section view of the transistor, the gate stack is clearly visible (see Figure 6d). From the diffraction patterns, which are shown for the silicon, silicon oxide, and hafnium oxide region in Figure 6a–c, respectively, the different materials can be assigned correctly as shown in Figure 6e and the HfO_2_ layer consists predominantly of the orthorhombic phase. Furthermore, the crystallographic orientation of the present grain along the gate stack is close to the [111]-axis (see Figure 6f).

## 4. Discussion

When comparing the HSO and HZO films (Figure 2d,e), the HSO layer seems to contain a higher fraction of monoclinic grains. By indexing the individual grains, the fraction of grains assigned to the monoclinic phase reaches 32.4% in case of HSO, whereas it is 3.8% for the HZO layer. This is further supported by the P–V loops, which showed a strongly decreased remanent polarization for HSO. Due to the large area of the measured capacitors, the measured polarization resembles an average value of all included grains. Differences in the exact fraction for TKD and P–V loops can be explained by differences in texture of the two layers and TKD representing a fraction of the grains, whereas P–V loops represent a fraction of the area. Additionally, GIXRD also agrees with these findings, as the monoclinic lines show a higher intensity.

The observed differences in the two materials could have various origins. One possible explanation is the dopant distribution inside the material. Zr, which is very similar to Hf regarding its chemical behavior and ionic radius (r_Zr_ = 0.84 Å, r_Hf_ = 0.83 Å) [18], shows a stabilization of the ferroelectric phase in HfO_2_ over a broad concentration distribution with a maximum at around 50 atm% [2]. Si, on the other hand, has a much smaller ionic radius (r_Si_ = 0.4 Å) than Hf [18] and already small concentrations around 3 atm% are enough to stabilize the orthorhombic phase [1]. Therefore, small fluctuations of the silicon content inside the layer may result in different phases. Since the present Si concentration in the sample are slightly lower than the optimal concentration, monoclinic grains are expected. Another explanation is the larger grain size in the HSO film that can be seen in Figure 2f, which displays the cumulative distribution of the equivalent grain diameter for HZO and HSO. While the average equivalent diameter of the orthorhombic grains in the HZO film is 28.5 nm, it is 33.9 nm in the case of the HSO film. On the contrary, the average diameter for the monoclinic grains is similar for both films with 33.2 nm and 33.9 nm for HZO and HSO, respectively. Since larger grains have been suggested to favor the monoclinic phase stronger as well as having a higher phase transition temperature for tetragonal to monoclinic [19], thus being less hindered by kinetic factors during this transition, a higher monoclinic phase fraction appears reasonable. The origin for larger grains in the HSO layer could be of interest for future research, as they are not expected due to the higher crystallization temperature of HSO [1,20].

The resulting device influences from the increased monoclinic phase can be seen in the in-die variability of the FeFETs. While the large devices show a rather low variability, the small ones show a very broad MW distribution. Some of them, probably those which consist mainly of monoclinic grains, are not even functional. This broader distribution can be assigned to a higher probability for different phase fractions inside the transistor area. Larger devices are creating an average over a larger area, thus showing a smaller distribution. In the case of HZO, the variability is smaller, as there are only very few monoclinic grains. Furthermore, due to the smaller grain size in HZO, the devices average over a larger amount of grains, improving variability further. An improved variability of the HSO due to a strong texture as suggested by the other measurements cannot be detected, as it is either superimposed by the phase variability or not present due to a different stack structure compared to MFM. In a purely orthorhombic layer, a pronounced texture would result in a narrow coercive field and remanent polarization distribution, which would improve device variability further, as the memory window depends on these two parameters. Process improvements as well as slightly higher silicon content, which favors less monoclinic phase, could counteract this problem.

**Figure 7:**
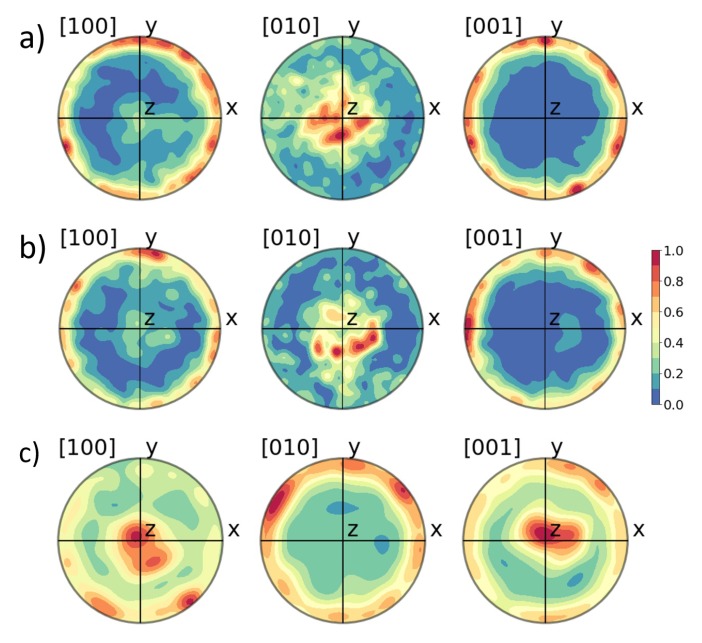
Shown are the pole figures of the [100]-, [010]-, and [001]-axes for orhorhombic grains in HZO (**a**), in HSO (**b**), and for monoclinic grains in HSO (**c**).

**Figure 8:**
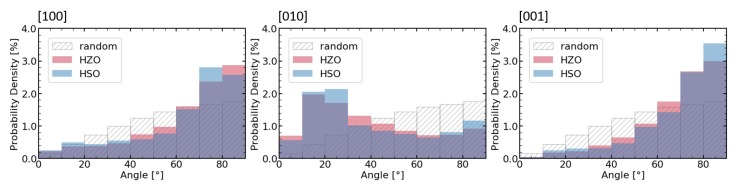
Textures of the <100>-axes are visualized by the angle distribution of the axis and sample normal. For comparison, the distribution of 100,000 randomly oriented vectors is simulated.

TKD and GIXRD as well as electrical measurements suggested a strong out-of-plane texture of the [010]-axis or even a preferred crystallographic orientation for the orthorhombic grains inside the HSO layer. This can also be seen in the pole figures (see Figure 7a). The pole figures of the HZO layer (see Figure 7b), as well as previously reported TKD analysis of the HZO film [15], have shown a similar texture. In order to compare the orientation of these two differently doped thin films, the distribution of the angle between the [001]-, [010]-, and [001]-axes and the thin film normal are extracted from the crystallographic orientation maps (Figure 3) of the doped hafnium oxide layers and compared in Figure 8. The texture of both films appear here very clearly in comparison to the simulated angle distribution for randomly oriented grains. While the [010]-axis favors an orientation parallel to the sample normal, the other two axes lie favorably in-plane. Furthermore, this texture is more strongly pronounced in the HSO sample for all directions.

This is further confirmed by GIXRD, which measures the diffraction pattern close to the in-plane orientation, revealing an increased intensity for the [100]-axis as well as <200>-axes. Additionally, it agrees with a stronger pronounced texture in the case of HSO. Furthermore, a [010] out-of-plane texture is supported by an initially pinched P–V loop, which opens during cycling due to ferroelastic switching [8,15]. The stronger textured HSO is further apparent in the very sharp switching peak in the I–V loop after wake-up.

The origin for a [010] out-of-plane texture has been suggested to be tensile in-plane stress in the thin film during crystallization and phase transition upon cool down due to the capping layer, in this case a TiN electrode, and differences in thermal expansion of the materials in the stack [15,21]. Furthermore, the precursor chemistry could influence the material behavior. The stronger pronounced texture in the case of HSO, while having the same processing parameters like HZO, can be attributed to differences of the thermal expansion coefficient and other material parameters due to the doping, as Si doping tends to favor shorter dopant-oxygen bonds [22]. Furthermore, the crystallization temperature for HSO (above 500 °C [1]) lies at higher temperatures than HZO, which crystallizes already at around 400 °C [20]. Therefore, nucleation and grain growth will occur at different temperatures for the two materials, which becomes also apparent in differences in the grain size and phase distribution discussed above.

In the case of the monoclinic phase, the GIXRD emphasized a [100] texture. From the pole figures in Figure 7c, a preferred orientation of the <100>-axes either close to the in-plane or out-of-plane directions is apparent. Only a few measurement points showed an orientation around 45° to the sample plane, thus agreeing with the low intensity of the diffraction lines at around 25° or 30°.

For the discussion of the structural analysis of the highly scaled FeFET, the planar view (see Figure 5) is considered first. The monoclinic fraction appears to be lower compared to the HSO MFM sample. This is explainable by the different stack structure of the two samples. The sample analyzed by TKD was a metal-ferroelectric-metal structure, whereas the STEM sample is a metal-ferroelectric-insulator-semiconductor (MFIS) structure. Therefore, the HfO_2_ is crystallizing on top of a TiN electrode or SiO_2_ interface layer, respectively. This has strong influences on the strain conditions inside the stack, as well as it being able to affect the deposition process and the nucleation.

Additionally, the pronounced texture as for the MFM sample cannot be detected, thus indicating that the stress conditions in this stack are different and probably lower in case of the MFIS sample. Therefore, it demonstrates that MFM stacks do not suffice for material development of HfO_2_ for the application in integrated FeFETs or rather the necessity for further research regarding the material behavior in MFIS stacks.

Finally, the large area crystallographic phase and orientation analysis of an integrated HfO_2_ based FeFET using STEM techniques has been demonstrated. The cross-section of the 22 nm FDSOI FeFET showed an orthorhombic HfO_2_ layer with an [111] orientation along the gate stack. This orientation has been reported to be favorable for the application as non-volatile memory device, as less electrical field drops across the interface layer, therefore improving endurance and retention [23].

## 5. Conclusions

In conclusion, it was demonstrated that TKD enables more detailed insight in the microstructure of ultra-thin films. Especially, it enables analyzing grain size, phase, and orientation distributions. Supported by electrical measurements and XRD, it could be shown that Silicon doped HfO_2_ results in a stronger textured film than HZO, mainly attributed to the stress conditions inside the layer stack. Furthermore, an accurate phase fraction of the monoclinic phase could be extracted for both films, showing an increased monoclinic phase in the HSO layer for certain process conditions. In terms of the application in FeFETs, the influences of texture as well as phase distribution are discussed.

Secondly, the phase and orientation analysis of an integrated highly scaled 22 nm FDSOI FeFET utilizing a STEM with a 2D detector was demonstrated. With this technique, it was shown that the predominant phase in the HSO layer is the orthorhombic phase, and that the present crystallographic orientation in the FeFET was the [111]-axis, which is preferred for improving endurance and retention. These results also suggested that more research regarding MFIS stacks is required in the future.
